# Granulosa cells exposed to fibroblast growth factor 8 and 18 reveal early onset of cell growth and survival

**DOI:** 10.18502/ijrm.v17i6.4815

**Published:** 2019-07-29

**Authors:** Fatemeh Amin Marashi, Ali Torabi, Francis Beaudry

**Affiliations:** ^1^Centre de Recherche en Reproduction et Fertilité, Faculté de Médecine Vétérinaire, Université de Montréal, St-Hyacinthe, Québec, Canada J2S 7C6.; ^2^Groupe de Recherche en Pharmacologie Animale du Québec (GREPAQ), Département de Biomédecine Vétérinaire, Faculté de Médecine Vétérinaire, Université de Montréal, St-Hyacinthe, Québec, Canada.

**Keywords:** Fibroblast growth factors, Proteomics, Mass spectrometry, Granulosa cell, Proliferation.

## Abstract

**Background:**

Fibroblast growth factors (FGFs) are growth factors that have diverse biological activities including broad mitogenic and cell survival activities. They function through the activation of a specific tyrosine kinase receptor that transduces the signal by activating several intracellular signaling pathways.

**Objective:**

To identify the different signaling pathways involved in the mechanism of action of FGF8 and FGF18 on ovine granulosa cells using mass spectrometry.

**Materials and Methods:**

Ovine ovarian granulosa cells were harvested from adult sheep independently at the stage of the estrous cycle and were cultured at a density of 500,000 viable cells in 1 ml DMEM/F12 medium for five days. The cells were then treated on day 5 of culture with 10 ng/mL FGF8 and FGF18 for 30 minutes, and total cell protein was collected for mass spectrometry.

**Results:**

Mass spectrometry showed that both FGF8 and FGF18 significantly induce simultaneous upregulation of several proteins, including ATF1, STAT3, MAPK1, MAPK3, MAPK14, PLCG1, PLCG2, PKCA, PIK3CA, RAF1, GAB1, and BAG2 (> 1.5-fold; p < 0.01).

**Conclusion:**

ATF1 and STAT3 are important transcription factors involved in cell growth, proliferation and survival, and consequently can hamper or rescue the normal ovine reproductive system function.

## 1. Introduction

The fibroblast growth factors (FGFs) are growth factors that have diverse biological activities including broad mitogenic and cell survival activities (1). FGFs constitute a large family of 22 distinct polypeptide growth factors varying in size from 17 to 34 kDa (2). They play a fundamental role in the regulation of embryogenesis. More precisely, they are responsible for cell growth, differentiation, proliferation, and cell migration. FGFs play a fundamental role in several stages of follicular development from preantral to preovulatory stage. Specifically, FGFs have been identified to regulate the initiation of primordial follicle growth, granulosa, and theca cell proliferation, differentiation, angiogenesis, and steroidogenes (3). It is presumed that FGF8 and FGF18 have similar receptor activation patterns leading to similar actions in ovine granulosa cells (4). A recent study proposed that FGF8 and FGF18 increase follicular health by increasing proliferation and suppressing cell differentiation (5).

The FGF receptors (FGFRs) include four major receptors (FGFR1-4) that, like other receptor tyrosine kinase, are activated by specific ligands. FGFRs are composed of two or three Ig-like loops in the external domain, a transmembrane domain and a ligand-activated cytoplasmic tyrosine kinase domain (6, 7). The two membrane-proximal Ig loops (Ig-II and Ig-III) comprise the ligand binding domain, although alternative splicing of Ig-loop III can generate isoforms (III b and III c) with distinct ligand binding properties and tissue distributions (8). Binding of FGF and HSPG to the extracellular ligand domain of FGFR (FGFR1c, FGFR2c, FGFR3c, FGFR3b, and FGFR4) induces receptor dimerization, activation, and autophosphorylation of multiple tyrosine residues in the cytoplasmic domain of the receptor molecule (9). A variety of signaling proteins are phosphorylated in response to the FGF stimulation including Shc, PLCγ, STAT1, Gab1, and FRS2α leading to stimulation of intracellular signaling pathways that control cell proliferation, cell differentiation, cell migration, cell survival, and cell shape (10). The docking proteins FRS2α and FRS2β are major mediators of the Ras/MAPK and PI-3 kinase/Akt signaling pathways as well as negative feedback mechanisms that fine-tune the signal that is initiated at the cell surface following FGFR stimulation (11). This pathway is important in granulosa cells (12). Signal transducer and activator of transcription 3 (STAT3) was identified as a phospho-dependent partner for FGFRs. STAT3 has a key role in many cellular processes such as cell growth and apoptosis (13).

The objective of the present study was to compare the effects of FGF8 and FGF18 on cell growth using the ovine granulosa cell model. Since FGF8 and FGF18 are homologous factors that share similar amino acid sequence but also differences, we hypothesized that they may interact differently to FGF receptors (i.e. FGFR2 and FGFR3) leading to distinct effects on granulosa cell such as induction of proliferation when they are exposed to FGF8 or 18 for a short period of time. Thus, we compared at the proteome level the effects triggered by FGF8 or FGF18 on the activation of FGFRs on ovine granulosa cells. A bottom-up proteomic mass spectrometry with label-free quantification was used followed by bioinformatic analyses.

## 2. Materials and Methods

This study is a fundamental and in vitro research study, and all the materials used were collected from the slaughterhouses across Quebec province in 2015–2017. All materials for cell culture were obtained from Life Technologies Inc. (Thermo Fisher Scientific, Burlington, ON, Canada) unless otherwise stated. Ovine granulosa cells were cultured in serum-free conditions that maintain estradiol secretion and responsiveness to follicle-stimulating hormone (14). Ovine ovaries were obtained from adult sheep irrespective of the stage of estrous cycle, at an abattoir and transported to the laboratory at 30°C in phosphate-buffered saline containing penicillin (100 µg/mL), streptomycin (100 µg/mL), and fungizone (1 µg/mL). Granulosa cells were harvested from follicles 12 mm diameter, and the cell suspension was filtered through a 150-mesh steel sieve (sigma-Aldrich Canada, Oakville ON). Cell viability was assessed by trypan blue dye exclusion. Cells were seeded into 24-well tissue culture plates (Sarstedt Inc., Newton, Nc) at a density of 0.5 million viable cells in 1 mL DMEM/F12 containing sodium bicarbonate (10 mmol/L), sodium selenite (4 ng/mL), bovine serum albumin (BSA) (0.1%; Sigma-Aldrich), penicillin (100 µg/mL), streptomycin (100 µg/mL), transferrin (2.5 µg/mL), nonessential amino acid mix (1.1 mmol/L), bovine insulin (10 ng/mL), androstenedione (10${}^{7}$ M at the start of culture and 10${}^{6}$ M at each medium change), and bovine follicle-stimulating hormone (10 ng/mL) starting on day 2; AFP5346D; National hormone and peptide program, Torrance, CA). Cultures were maintained at 37°C in 5% CO${}_{2}$, 95% air for five days with 70% medium being replaced on days 2 and 4. To assess the effect of FGF8 and of FGF18 on intracellular pathway activation, cells were treated on day 5 of culture with 10 ng/mL recombinant human FGF8 and FGF18 (Pepro Tech) for 30 min, then cells were lysed and protein solubilized in RIPA buffer. All experiments were performed with three different pools of cells, each collected on different occasions. The samples were stored at –80°C pending mass spectrometry analysis. Proteins were extracted from cell samples and bottom-up proteomic analysis were performed. The total amount of protein in each sample was determined to use a standard Bradford assay. Briefly, a volume corresponding to 50 µg of proteins was used for each sample. Proteins were isolated using a precipitation procedure with a ratio 1:3 (v:v) of acetone. The samples were centrifuged at 9,000 g for 10 min. Then, acetone was discarded, and protein pellet was dried for 20 min in a vacuum centrifuge set at 60°C. The protein pellet was dissolved in 100 µL of 50 mM ammonium bicarbonate (pH = 8), and the solution was sonicated for 60 min at maximum intensity to improve and protein dissolution yield. Reduction and alkylation were performed as previously described (15), then, 2 µg of proteomic-grade trypsin was added, and the reaction was performed at 40°C for 24 hr. The protein digestion was quenched by adding 10 µL of a 2% TFA solution. Samples were centrifuged at 12,000 g for 10 min, and the supernatants were transferred into injection vials for analysis.

The HPLC system was a Thermo Scientific UltiMate 3000 Rapid Separation UHPLC system (San Jose, CA, USA). The chromatography was achieved using a gradient mobile phase along with a microbore column Thermo Biobasic C8 100×1 mm, with a particle size of 5 μm. The initial mobile phase condition consisted of acetonitrile and water (both fortified with 0.1% of formic acid) at a ratio of 5:95. From 0 to 1 min, the ratio was maintained at 5:95. From 1 to 61 min, a linear gradient was applied up to a ratio of 50:50 and maintained for 2 min. The mobile phase composition ratio was reverted at the initial conditions, and the column was allowed to re-equilibrate for 14 min for a total run time of 77 min. The flow rate was fixed at 75 µL/min, and 2 µL of samples were injected. A Thermo Scientific Q Exactive Orbitrap Mass Spectrometer (San Jose, CA, USA) was interfaced with a Thermo Scientific UltiMate 3000 Rapid Separation UHPLC system using a pneumatic assisted heated electrospray ion source. MS detection was performed in positive ion mode and operating in scan mode at high-resolution, and accurate-mass (HRAM). Nitrogen was used for sheath and auxiliary gases, and they were set at 10 and 5 arbitrary units. The ESI voltage was set to 4000 V and the ion transfer tube temperature was set to 300°C. The default scan range was set to m/z 4001500. Data was acquired at a resolving power of 140,000 (FWHM) using automatic gain control targets of 3.0×10${}^{6}$ and maximum ion injection time of 200 msec. Additionally, MS data were acquired using a data-dependent top-10 method to dynamically choose the most abundant precursor ions from the survey scans (400-1500 Da) and generate MS/MS spectra. Instrument calibration was performed prior to all analysis, and mass accuracy was notably below 1 ppm using Thermo Pierce calibration solution and automated instrument protocol.

### Ethical consideration

This research study was done using ovine ovarian samples obtained from slaughterhouses in Québec. These experiments were approved by the Animal Ethics Committee of the Faculty of Veterinary Medicine of the Université de Montréal.

### Statistical analysis

Database surveys were performed using Proteome Discoverer software (version 2.1) with Uniprot ovine protein database (extracted FASTA file). Mass tolerance of precursor and fragment (i.e., typically b and y) were set at 5 ppm and 10 ppm, respectively. Phosphorylation at Y and T amino acids was set as a variable post-translational modification. Quantification was based on MS${}^{1}$ ion intensity, and peptide identification was based on precursor ion (MS${}^{1}$) and at least three characteristic (MS${}^{2}$).

Label-free MS${}^{1}$ quantification of peptide/protein via peak intensity was performed using SIEVE (version 2.1), a label-free differential expression software that aligns the MS spectra over time from different data sets and then determines the structures in the data (m/z and retention time pairs) that differ. These differences were examined using statistical methods (e.g., p-value and standard deviation) and then sorted based on significance using the peak intensity values obtained from the data of each biological replicate. The p-values with four decimal significant digits are provided in Table I. The following parameters were set to align the retention time and generate the frames needed for abundance calculations. Alignment Parameters; Alignment Bypass = False, Correlation Bin Width = 1, RT Limits for Alignment = True, Tile size = 300, Max RT Shift = 0.2, m/z Min = 400, m/z Max = 1,500, Frame time Width (min) = 2.5 minutes, Frame m/z width = 10 ppm, Retention Time Start = 2.0 min, Retention Time Stop = 65 min, Peak Intensity threshold = 100,000. Significance was calculated within SIEVE using a standard *t*-test, and results were filtered using the identification criteria stated earlier. Statistical significance was set at a p-value < 0.01. Interactomic analyzes were performed using Genemania and STRING interfaces and databases.

## 3. Results 

**Figure 1:**
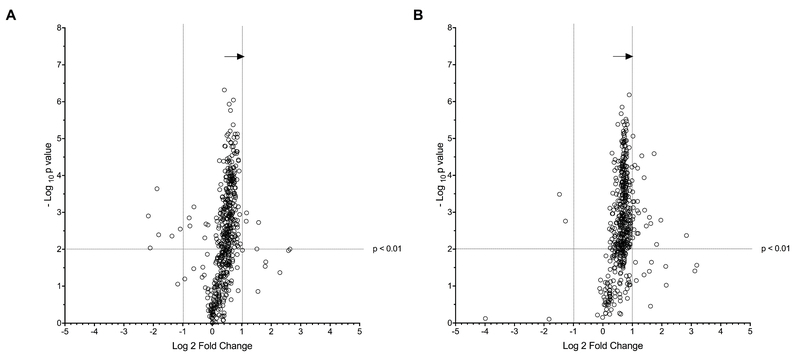
A volcano plot revealing differentially expressed proteins between control and FGF8 (A) as well as control versus FGF18 (B). As displayed, the exposition of ovine granulosa cells to FGF8 or FGF18 has led to a significant up-regulation of numerous proteins and very few were down-regulated. Also, significance was calculated within SIEVE using a standard *t*-test. Statistical significance was set at a p-value < 0.01.

Mass spectrometry showed that both FGF8 and FGF18 significantly induce simultaneous upregulation of several proteins, and Table I includes the most abundant proteins observed and validated based on spectral libraries and *in silico* bottom-up proteomic analyses (e.g., Proteome Discoverer-SEQUEST). A total of 32 up-regulated proteins (i.e., fold change > 1.5 for FGF8 or FGF18) were identified, as shown in Table I. Figure 1 exhibits a volcano plot revealing differentially expressed proteins between control and FGF8 (Figure 1A) as well as control versus FGF18 (Figure 1B). As displayed, the exposition of ovine granulosa cells to FGF8 or FGF18 has led to a significant up-regulation of numerous proteins, and very few were down-regulated. Moreover, very few proteins were unaffected by either FGF8 or FGF18. This observation is coherent with an early onset of cell growth and proliferation, an expected outcome since FGFR signaling pathways are associated with both. Additionally, despite it not being statistically significant, we observed a more important up-regulation following the exposition to FGF18 compared to FGF8.

In Table I, the significance was calculated within SIEVE using a standard *t*-test and results were filtered using the identification criteria stated earlier. These differences were examined using statistical methods (e.g., p-value and standard deviation) and then sorted based on significance using the peak intensity values obtained from the data of each biological replicate (control, FGF8, and FGF18). Statistical significance was set at a p < 0.01.

**Table 1:** Proteins whose level of phosphorylation was increased in response to FGF8 and FGF18 in ovine ovarian granulosa cells


**Protein**	**Accession No**	**FGF8**		**FGF18**	
	**Fold change (SD)**	**P-value**	**Fold change (SD) **	**P-value**
CAMK2A	Q9UQM7	1.41 (0.056)	0.0001	1.56 (0.067)	0.0002
YWHAB	P31946	1.23 (0.042)	0.0170	1.58 (0.065)	0.0001
GRB10	Q13322	1.29 (0.030)	0.0040	1.59 (0.040)	0.0001
TRADD	Q15628	1.25 (0.035)	0.0117	1.60 (0.040)	0.0018
EPHA5	P54756	1.40 (0.086)	0.0217	1.62 (0.086)	0.0040
PLCG2	P16885	1.35 (0.042)	0.0002	1.64 (0.051)	0.0070
IRF2BP1	Q8IU81	1.48 (0.042)	0.0174	1.64 (0.062)	0.024
PIK3CA	P42336	1.40 (0.030)	0.0126	1.65 (0.044)	0.0024
YWHAE	P6225	1.58 (0.025)	0.0017	1.67 (0.046)	0.009
STAT3	P40763	1.54 (0.058)	0.0001	1.67 (0.065)	0.001
IGF1	Q00997	1.46 (0.04)	0.0003	1.69 (0.054)	0.0002
MDM2	Q00987	1.47 (0.075)	0.0034	1.71 (0.076)	0.022
PLCG1	P19147	1.68 (0.182)	0.0222	1.71 (0.206)	0.0151
ATK1	O00139	1.57 (0.080)	0.0007	1.71 (0.098)	0.0073
KRAS	P01116	1.44 (0.098)	0.042	1.71 (0.160)	0.006
MAPK14	Q16539	1.28 (0.055)	0.0001	1.72 (0.081)	0.0007
MAPK3	P27361	1.79 (0.141)	0.014	1.76 (0.074)	0.0003
MAP2K1	Q02750	1.29 (0.291)	0.8360	1.77 (0.348)	0.12
HRAS	P01112	1.35 (0.162)	0.0416	1.78 (0.229)	0.0515
MAPK1	P28482	1.88 (0.037)	0.0001	2.01 (0.043)	0.0003
PGRMC1	O00264	1.85 (0.062)	0.062	2.02 (0.118)	0.005
CTSA	P10619	1.68 (0.081)	0.0001	2.04 (0.121)	0.0007
GPS2	Q13227	1.45 (0.138)	0.0070	2.08 (0.203)	0.003
XDH	P47989	1.68 (0.086)	0.0003	2.12 (0.102)	0.0001
RASGRP3	Q81V61	1.58 (0.177)	0.006	2.32 (0.275)	0.0010
GAB1	Q13480	1.79 (0.263)	0.0052	2.67 (0.371)	0.0009
RAF1	P04049	2.07 (0.510)	0.0042	2.75 (0.645)	0.0042
BAG2	O95816	2.03 (0.881)	0.0106	3.07 (1.069)	0.351
SNX3	O60493	1.78 (0.408)	0.0065	3.09 (0.434)	0.002
SF3A3	Q12874	3.47 (1.316)	0.030	4.39 (1.648)	0.003
PKCA	P17252	3.52 (1.581)	0.022	4.44 (1.764)	0.095
ATF1	P18846	6.11 (2.983)	0.010	8.90 (3.793)	0.028
**** ***CAMK2A:*** **** Calcium/calmodulin-dependent protein kinase type II alpha chain (CAMKIIα); **** ***YWHAB:*** **** 14-3-3 protein beta/alpha; **** ***GRB10:*** **** Growth factor receptor-bound protein 10; **** ***TRADD:*** **** Tumor necrosis factor receptor type 1-associated DEATH domain protein; **** ***EPHA5:*** **** EPH receptor A5 (ephrin type-A receptor 5); **** ***PLCG2:*** **** 1-Phosphatidylinositol-4, 5-bisphosphate phosphodiesterase gamma-2; **** ***IRF2BP1:*** **** Interferon regulatory factor 2 binding protein 1; **** ***PIK3CA:*** **** phosphatidylinositol-4, 5-bisphosphate 3-kinase, catalytic subunit alpha; **** ***YWHAE:*** **** 14-3-3 protein epsilon; **** ***STAT3:*** **** Signal transducer and activator of transcription 3; **** ***IGF1:*** **** Insulin-like growth factor 1;**** *** MDM2:*** **** Mouse double minute 2 homolog; **** ***PLCG1:*** **** Phospholipase C, gamma 1; **** ***ATK1:*** **** kinesin like protein in Arabidopsis thaliana; **** ***KRAS:*** **** Kirsten rat sarcoma; **** ***MAPK14:*** **** Mitogen-activated protein kinase 14; **** ***MAPK3:*** **** Mitogen-activated protein kinase 3; **** ***HRAS:*** **** GTPase HRas also known as transforming protein p21; **** ***PGRMC1:*** **** Progesterone receptor membrane component 1; **** ***CTSA:*** **** Cathepsin A; **** ***GPS2:*** **** G protein pathway suppressor 2; **** ***XDH:*** **** Xanthine dehydrogenase; **** ***RASGRP3:*** **** Ras guanyl-releasing protein 3; **** ***GAB1:*** **** GRB2-associated-binding protein 1; **** ***RAF1:*** **** Rapidly accelerated fibrosarcoma; **** ***BAG2:*** **** BAG family molecular chaperone regulator 2; **** ***SNX3:*** **** Sorting nexin-3; **** ***SF3A3:*** **** Splicing factor 3A subunit 3; **** ***PKCA:*** **** Protein kinase c; **** ***ATF1:*** **** Cyclic AMP-dependent transcription factor.					

## 4. Discussion

This observation is compatible with our initial hypothesis. FGF8 and FGF18 are homologous growth factors that possess similar sequence homology, but they appear to interact with FGFRs differently leading to a more pronounce growth or proliferation effect that may lead to a distinct outcome for granulosa cells.

**Figure 2:**
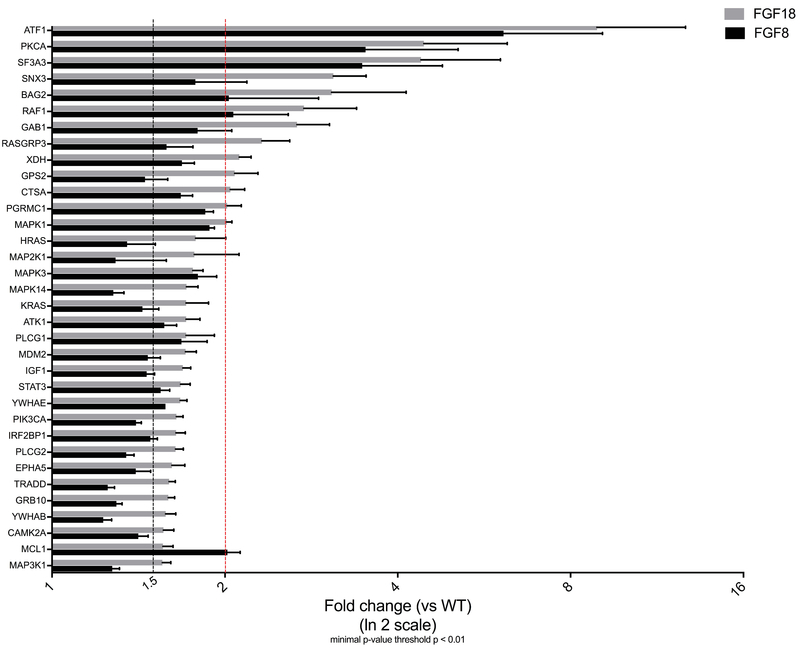
It is a graphical representation of Table I.

Interestingly, two very important transcription factors were identified, STAT3 and ATF1. STAT3 is a member of the STAT family that was identified as a DNA-binding factor (4, 16) and is expressed early during post-implantation in most tissues. Moreover, STAT3${}^{-/-}$ mice are characterized by embryonic lethality. STAT3 is involved in promoting cell growth and is constitutively active and potentiate tumorigenesis (17, 18). Pro-oncogenic STAT3 activity is linked with gene expression that is known to promote proliferation and inhibit apoptosis (19). Several serine kinases have been reported to be involved in the serine phosphorylation of STATs, including MAPK14 and MAPK1/3 that were also up-regulated following the exposition of granulosa cell to FGF8 and FGF18 for 30 minutes. With the advances in proteomic science, STAT3 was identified as an important molecule that plays numerous roles in cells. It has a two-fold role as a mediator of signaling and regulator of gene expression. Furthermore, STAT3 has been widely described as an oncogene correlated with tumor progression and up-regulated in many tumor specimens (20). Consequently, STAT3 signaling pathway could be considered as a promising therapeutic target. As shown in Table I and Figure 2, many members of the MAPK/ERK pathway were significantly up-regulated following the exposition of granulosa cells to FGF8 and FGF18 for 30 min. Growth factors and mitogens use the MAPK/ERK pathway signaling cascade to signal the regulation of gene expression and prevent apoptosis. Some constituents of these pathways are mutated or abnormally expressed in cancer. However, it may be advantageous to induce MAPK/ERK pathway expression to promote cell cycle arrest to stop deregulated cell proliferation. Activation of transcription factor 1 (ATF1) was significantly up-regulated following the exposition of granulosa cells to FGF8 and FGF18 for 30 minutes. ATF1 and RAS proteins were found significantly elevated in various tumors and were recently identified as potential clinical diagnostic biomarkers (21). Also, an important aspect is that ATF1 is up-regulated upon stress as part of a cell adaptation mechanism (22). ATF1 and CREB interact and are important for cell survival, particularly during early development. The extensive up-regulation of ATF1 could be a consequence of the onset of STAT3 promoting cell growth (23).

**Figure 3:**
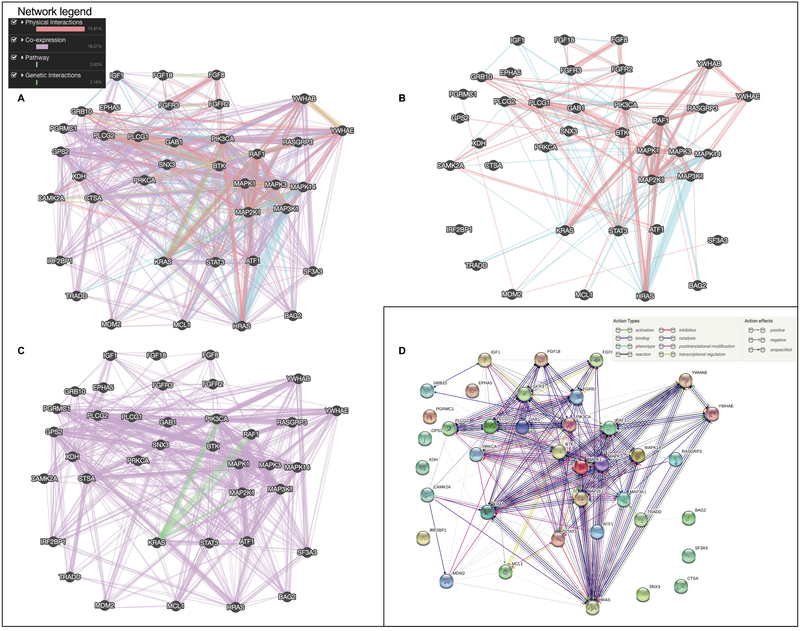
The interactomic analysis were performed using STRING interfaces and databases (this is not a statistical study).

Analysis of the STAT3 and ATF1 interactome (Genemania interface and database) shown in Figure 3 outline the important genetic interactions and co-expression (Figures 3A and 3C) for all the abundant proteins observed and presented in Table I and Figure 2. There is a strong genetic association between STAT3 and ATF1 but a minor physical or pathway association. This observation is also validated with a STRING analysis (i.e., predicted protein–protein interactions) in Figure 3D. This is coherent with our hypothesis that ATF1 might counterbalance the STAT3-induced cell growth, proliferation, and potentiation of tumorigenesis using an independent pathway. The interactomic analyses performed with Genemania and STRING presented in Figure 3 confirm a high degree of genetic and physical interactions between MAPK/ERK pathway signaling cascade with STAT3 and ATF1, which is coherent with symmetrical up-regulation observed following the exposition of granulosa cell to FGF8 and FGF18 for 30 min.

## 5. Conclusion

In summary, in ovine granulosa cells, FGF8 and FGF18 induced a significant up-regulation of numerous proteins associated with the MAPK/ERK pathway. More importantly, two important transcription factors were significantly up-regulated, STAT3 and ATF1, both interacting strongly with the MAPK/ERK pathway. The findings from this study described for the first time the FGF8 and FGF18 up-regulation of ATF1 and STAT3 expression in granulosa cells. Interestingly, STAT3 and ATF1 are involved in promoting cell growth and proliferation, and constitutively active STAT3 is able to potentiate tumorigenesis.

##  Conflict of Interest

No conflict of interests.
